# Insights into the Novel Therapeutics and Vaccines against Herpes Simplex Virus

**DOI:** 10.3390/vaccines11020325

**Published:** 2023-01-31

**Authors:** Shiza Malik, Ranjit Sah, Omar Ahsan, Khalid Muhammad, Yasir Waheed

**Affiliations:** 1 Bridging Health Foundation, Rawalpindi 46000, Pakistan; 2 Department of Microbiology, Institute of Medicine, Tribhuvan University Teaching Hospital, Kathmandu 44600, Nepal; 3 Department of Microbiology, Dr. D. Y. Patil Medical College, Hospital and Research Center, Dr. D. Y. Patil Vidyapeeth, Pune 411018, Maharashtra, India; 4 Department of Medicine, School of Health Sciences, Foundation University Islamabad, DHA Phase I, Islamabad 44000, Pakistan; 5 Department of Biology, College of Science, UAE University, Al Ain 15551, United Arab Emirates; 6 Office of Research, Innovation, and Commercialization (ORIC), Shaheed Zulfiqar Ali Bhutto Medical University, Islamabad 44000, Pakistan; 7 Gilbert and Rose-Marie Chagoury School of Medicine, Lebanese American University, Byblos 1401, Lebanon

**Keywords:** herpes simplex virus, HSV biology and infection, therapeutics, antiviral agents, vaccines, therapies, treatment, novel therapeutic, clinical management

## Abstract

Herpes simplex virus (HSV) is a great concern of the global health community due to its linked infection of inconspicuous nature and resultant serious medical consequences. Seropositive patients may develop ocular disease or genital herpes as characteristic infectious outcomes. Moreover, the infectious nature of HSV is so complex that the available therapeutic options have been modified in certain ways to cure it. However, no permanent and highly effective cure has been discovered. This review generates insights into the available prophylactic and therapeutic interventions against HSV. A methodological research approach is used for study design and data complication. Only the latest data from publications are acquired to shed light on updated therapeutic approaches. These studies indicate that the current antiviral therapeutics can suppress the symptoms and control viral transmission up to a certain level, but cannot eradicate the natural HSV infection and latency outcomes. Most trials that have entered the clinical phase are made part of this review to understand what is new within the field. Some vaccination approaches are also discussed. Moreover, some novel therapeutic options that are currently in research annals are given due consideration for future development. The data can enable the scientific community to direct their efforts to fill the gaps that remain unfilled in terms of therapies for HSV. The need is to integrate scientific efforts to produce a proper cure against HSV to control the virus spread, resistance, and mutation in future disease management.

## 1. Introduction

Herpes simplex virus (HSV) belongs to the family of Alphaherpesvirinae with a characteristic double-stranded DNA structural composition [1]. Its two main serotypes, HSV-1 and HSV-2, are mainly known for their links with infectious diseases [2]. According to estimates by WHO, approximately 70–90% of the population worldwide is seropositive for HSV-2, which makes it a great concern for the healthcare community regarding the possibility of developing infections (James & Kimberlin, 2015b) [3]. HSV-1 is considered the main causative agent of ocular infection that may occur in patients already having diseases such as genital lesions, keratitis or retinal necrosis, encephalitis, iridocyclitis, or conjunctivitis [4,5,6]. Some studies have also established links between longstanding HSV-1 serology with psychological complications, including Alzheimer’s disease [7]. On the other hand, HSV-2 is predominantly linked with characteristic genital herpes disease of the herpes virus. These infections are worldwide, irrespective of the developing or industrialized national standing [8]. 

HSV-2 virus is a sexually transmitted infectious agent that is prevalent in approximately 536 million people worldwide, standing at an annual incidence rate of about 23.6 million cases, as per the updates by the CDC [9,10]. The genital area is the prime target of viral infection; however, it may also cause infections similar to HSV-1, including necrotizing stromal keratitis in the eyes, meningitis, encephalitis, and neurological complications [11]. However, not all HSV-2-positive cases develop genital herpes or ulcerative and vesicular lesions because the virus mostly remains in a latency phase that makes it possible to transmit to other members of the populace without getting noticed and without exhibiting any infectious outcomes [12]. In simple words, the sexual transmission may not be coupled with a clinical history or symptomatology of genital herpes. The symptomology makes the viral presence rarely fatal, but the same is not the case for babies from infected mothers and pregnant ladies, due to their immunocompromised system and susceptibility to easily acquire infection [12,13,14]. 

The most prevalent feature that makes HSV infection complications is its ability to enter a nonreplicating latency period, which enables it to survive long periods of inactivation within the host and gives it the ability to reactivate and infect the host under different external and internal stimuli [12,13]. The latency period is mostly asymptomatic; thus, viral transmission during this period remains unrecognized. This aspect is considered the major reason for the large-scale seroprevalence of HSV [13]. Additionally, it can infect almost every type of cell for the characteristic receptor recognition strategies, making it pertinent to large-scale population spread [10]. This property is associated with the presence of hundreds of diverse glycoproteins in HSV lipid bilayers that alter its receptor recognition and viral entry into host cells [8,15,16,17,18,19]. Furthermore, it uses multiple strategies for viral fusion, endocytosis, and transmission among cells, all of which cause further complications its treatment procedures [20,21]. Thus, most of the therapeutic studies are limited in terms of their effectiveness and inability to eradicate infections. 

The complex nature of the virus, in terms of symptomatic infections and asymptomatic presence and recurrence, makes its cure difficult; for this reason, no vaccination or therapeutic cure has been devised that could completely eradicate HSV infection [21,22]. Moreover, the lifelong presence of HSV-2 infection further complicates the treatment procedures because it may require prolonged administration of standard proposed treatments, which is in line with its resistive nature [12,23]. In this review, we discuss the primary nature of HSV that makes it complicated for the design of therapeutics. This understanding can set the pace for further insight into the therapeutic interventions designed to date [24]. We discuss how the antiviral strategies are designed knowing the complexities associated with viral entry, infection, and persistence. The focus is on therapeutic developments that may hold hope for future control over HSV infections. 

## 2. Materials and Methods

A systematic approach was used to gather the latest data regarding the different dimensions of H. simplex virus infection and the accumulated therapeutic and vaccination strategies. We searched electronic sources such as Google Scholar, Pub Med, NIH (National Library of Medicine), Scopus (Elsevier), and Web of Science. Moreover, the official websites of WHO, CDC, UNAID, and FDA were also used to obtain the statistical results and latest updates regarding HSV epidemiology, as well as ongoing treatment efforts. As the studies mainly incorporated the data regarding therapeutics against HSV viruses, the major research terms were “Herpes simplex virus”, “HSV infection”, “therapeutics against HSV”, “antiviral agents”, “vaccines against HSV”, “therapies against HSV”, “novel therapeutic approaches”, and some other linked search terms. After a thorough analysis of the dates, abstracts, titles, and journals of research publications, they were made part of this review. The process of information gathering was not limited to a few studies but rather collected from research compilations in the form of original research articles, reviews, short commentaries, case reports, and letters to the editors. Lastly, the search strategy was limited to incorporating data from 2010 to 2022 to add only the most recent advances related to HSV disease management.

## 3. Results and Discussion

### 3.1. The Pathological Biology of HSV and Associated Infection

As explained earlier, HSV belongs to the family of neurotropic alpha herpesviruses, which are well known for latency features [4,12]. The virus particle consists of an internal dense electron core that contains the reproductive material in the form of double-stranded DNA [25]. All age groups of people are prone to developing HSV infection. Some other pathogenic species of viruses belonging to the same DNA herpes family of viruses include varicella-zoster virus (VZV), Epstein–Barr virus (EBV), cytomegalovirus, and human herpes types 6, 7, and 8 [26]. The two subtypes of HSV-1 and HSV-2 share close genomic relevance with >80% of the amino-acid identity profile. Moreover, both share the common infectious nature of causing oral and genital ulcerations [26,27].

HSV DNA has been well studied and formulated to encode 70+ genes. The genomic portion is enveloped by a viral capsid of icosahedral shape that in turn displays >162 known protein units (capsomers) [28,29]. The virus capsid is further surrounded by a lipid bilayer envelope consisting of tegument proteins and several dozen glycoproteins on the surface. Of this GP, five glycoproteins with known functions are gB, gC, gD, gH, and gl [30]. These proteins facilitate host viral coordination in terms of attachment, binding, and host cell penetration and entry [31,32]. The complex mechanism of HSV host interaction and its latency period present scientists with a great challenge [33]. The host virus entry mechanisms, cellular interaction, and infectious cycles are, thus, the prime focus of understanding for scientists developing therapeutics. This is discussed in relevance to HSV therapeutics in a later section of this article to describe to the readers the procedures, limitations, and progress achieved in this domain [27,34]. 

The virus Infection mainly begins with the epithelial cells of the skin or mucosal surfaces. The virus particle then trickles down to nerve endings and nerve axons, where the virus undergoes persistent infection within the trigeminal or lumbosacral ganglia region [12,35]. After establishing virus progeny in this area, it returns to the mucosal and skin surfaces to produce oral or genital ulcers. At other times, it remains asymptomatic, associated with viral shedding and silent transmission to other hosts [36,37]. This asymptomatic and silent transmission nature makes HSV spread quite extensively unnoticed in the population, as most cases remain subclinical and, thus, hidden from diagnosis [1,21]. The clinical manifestations of HSV-1 and HSV-2 vary depending on the age group, entry route, host immune response, and initial or recurrent nature of the infection [1]. 

HSV-1 is linked with episodes of genital and neonatal herpes, in addition to mainly causing oral and facial infections [38]. The disease incidences are higher in HICs, though the disease occurrence rates in neonates are lower compared to the incidence in children in LICs, where ≥90% people acquire HSV-1 infection by adolescence, which makes it a great healthcare burden for the world [10]. The major disease outcomes of HSV-1 infection include herpes labialis, gingivostomatitis, HSV-linked infectious encephalitis, keratitis, and pharyngitis [39]

HSV-2, on the other hand, is mainly dependent on sexual transmission and is associated with genital ulceration (genital ulcer disease (GUD)). Apart from the risk of neonatal herpes and possibly being associated with the development of neurological disorders such as Alzheimer’s at a later age, an increased risk of developing HIV infection is linked to HSV-2 infection [1,12,14]. The disease incidence is continuously rising on an annual basis due to the silent nature among sexual partners with high HSV-2 seroprevalence [3,13]. Additionally, the incidence rates are mostly outlined for high-income countries where R&D is progressively outlined in databases, while, in the case of low-income countries, the incidence rates are still unknown [10]. Neonatal herpes infections are often the major causes of increased morbidity and mortality rates. Most importantly, HSV-2 infection and transmission are linked with up to threefold increased incidence of HIV epidemics, with incidence rates up to 23–50% being the major concern regarding healthcare management. Moreover, the clinical complications in terms of life-threatening incidences may be accounted for in immunocompromised individuals [40]. 

The major diagnostic tests performed for HSV detection mainly include polymerase chain reaction, type-specific serological essays, viral culture, and antigen detection assays, which differentiate between the two subtypes of HSV [21,41]. The main treatment strategies against genital herpes mainly include antiviral treatments, such as acyclovir, valacyclovir, or famciclovir among other antiviral agents mentioned by WHO guidelines [3,9,22]. Most of these treatment regimens require continuous application to reduce the symptoms without permanently dealing with the virus prevalence. In the upcoming sections, we discuss the various therapeutic strategies and vaccination efforts against HSV carried out recently, as well as outline the futuristic perspective for HSV treatment. 

### 3.2. Current Vaccination Efforts against HSV

Currently, no vaccine has been specified against HSV infection; however, several vaccination candidates are in research annals for vaccine development. Most of the clinical efforts are directed toward HSV-2 for its greater infectious outcomes, but HSV-2 vaccines will have benefits against both subtypes of viruses because of their sequence homology [42,43,44]. Several lines of research show that HSV vaccination is feasible. Successful vaccination strategies against varicella-zoster virus by using replated herpes virus biology, bovine herpesvirus-1, and herpesvirus-1 (pseudorabies virus) indicate that effective vaccine efforts against HSV can be successful [44]. Similarly, work on vaccines along with antiviral adjuvants has also presented preventive abilities against herpes zoster infection, with proven 97% efficacy in phase III trials [43,44,45].

Studies on human papillomavirus vaccination protocols are also helping with the understanding of immunomodulatory regulation which can be effectively induced with vaccination against HSV [43,46]. Some other research groups tested herpes vaccine trials with truncated glycoprotein D2 (Gd2T) vaccines tested on thousands of HSV-positive cases and demonstrated vaccine efficacy of up to 58% against HSV-1 with little or no effect against HSV-2. These studies indicated that immunomodulation against HSV-1 can be achieved with antibody titers, but the same cannot yet be shown for HSV-2 [21,30]. Additionally, the viral sequencing, widescale data availability, and genetic profiling for both HSV-1 and HSV-2 predict that better vaccination protocols could be implemented with the identification of potential targets for therapeutic development. With proper and coordinated efforts among bioinformaticians and clinicians, these efforts could be successful [46,47].

The rigorous efforts put forward for vaccination development come in two major forms, preventive and therapeutic vaccination protocols [48]. The former provides pre-exposure immune-protective responses against HSV-2 infection development tested over the long term up to phase III trials [43]. These trials have mostly been limited to HIC. It is hypothesized that the same preventive vaccination practices in LMIC could produce better results in terms of disease mitigation and adaptive management. For this cause, the geographic strain diversity must be accounted for during vaccination development to ensure the nonrestrictive geographic nature of HSV vaccination. Scientists propose that, if any candidate vaccine was found to be effective for both HSV subtypes, it could be shifted from adolescents to infants for the possible prevention option [14,49].

Therapeutic vaccines, in contrast, are being tested to reduce the disease symptoms and viral transmission across the hosts, for the larger benefit of public health management. For this route, the targeted populace is mostly HSV-2-infected persons. Some therapeutic candidates have been checked up to phase I and II trials with proven antiviral effects, as well as decreased viral shedding and lesion formation in infected subjects [30,48]. Similar to the first line of preventive vaccinations, this route has also been tested mostly in HIC without solid testing in LMICs [20]; a limitation to these preclinical trials on animals is that they cannot be assumed to be effective in humans since the host viral interaction could be different in people. Thus, certain limitations are attached to phase I and II trials. Therefore, some effort should be put into clinical experimentation for rapid disease management in the future [47].

### 3.3. Outlining the Current Vaccines against HSV

Various vaccine candidates are in research annals for preclinical and clinical evaluation. Currently, there is no specific FDA-approved vaccine against HSV infection [9]. The ongoing clinical and preclinical trials are based on the rational understanding of HSV biology and immunopathogenesis in host cells. Some of the latest vaccine designs, their working mechanism, and ongoing trials are outlined briefly in the Table 1.

#### 3.3.1. Subunit Vaccines against HSV

Subunit vaccines are composed of viral components, such as glycoproteins and protein subunits, which undergo protective immune responses to the host [50]. They have proven safer, stable, and effective for HPV vaccination design and immunization design, but still lack clinical experimental success against HSV [51]. They mostly use viral glycoproteins and antigenic mediators such as Gb/Gd/gE in their antiviral design. This type of vaccine varies in function and procures the inhibition of viral entry, viral shedding, transmission across cells, and immune-evasive responses [42,50,51]. Novel experiments are ongoing that link multiple herpes antigens and peptide epitopes in one vaccination protocol. Approximately 80–300 open readings frames (ORFs) identified by multi-omics technologies are under consideration for antigenic breadth generation and efficiency in subunit vaccines against HSV [42,52,53].

This method of vaccination provides a gateway to present complex antigenic composition to the immune system that may include T- and B-cell epitopes [22,72]. For testing the efficacy of these vaccines, several recombinant protein formats have been tested that are conceptually similar and undergo the introduction of HSV ORFs (complete or near complete), into bacterial or other vector systems [54,73]. Moreover, these vaccine combinations with certain adjuvants and vaccine formats have opened a new route to explore options for HSV vaccination in the future.

#### 3.3.2. Vectored/DNA/RNA Vaccines against HSV

DNA- and mRNA-based vaccines have been in research annals for a long time now. The same approach has been successfully used in COVID vaccination design and is now being successfully utilized against HSV [74,75]. Experiments have been conducted on animal models to check the efficacy of nucleoside-modified mRNA-based vaccines against HSV-2 infection [75]. The results indicated a therapeutic reduction in symptoms within animal models in a dose-dependent manner. Moreover, these vaccines stimulate immune responses in the form of neutralizing antibodies [76]. Studies have shown that DNA is a better candidate for its stability, synthesis characteristics, and purification protocol and can be better managed compared to mRNA [29,62].

DNA-based vector vaccines have shown efficacy even better than subunit vaccines but not as effective as live-attenuated vaccines [29,62]. Moreover, some clinical concerns in the form of side-effects remain linked to the application of vehicle vector carriers [77]. Thus, adenovirus vector-based vaccines exhibit a better stability profile than mRNA vaccines. Recent studies showed the use of Vaccinia and MVA vectors for the deployment of transgenetic expression and virulence in tested subjects against different viral diseases caused by HIV, influenza, measles, flavivirus, and malaria vectors [11,45]. Thus, these insights into vaccination approaches compel scientists to drain effective vaccination efforts against HSV [78].

#### 3.3.3. Live-Attenuated Vaccines against HSV

The live-attenuated vaccination method has been the most used and effective method against viral infections through history, such as smallpox vaccination, poliomyelitis, measles, mumps, rubella virus, rotavirus, and many other infections [63]. The mechanism of inactivation often includes chemical or radiation-based inactivation of virus particles. One of the antiviral vaccine candidates derived for chicken pox virus/HSV-3 (varicella-zoster virus (VZV)) is also based on a live-attenuated virus vaccination protocol [62]. It is safe and well tolerated with a highly effective profile that controls viral reactivation. This and several other examples guide a more effective vaccination protocol to be designed on the basis of this mechanism [43,62,63,64].

Live-attenuated vaccination has also contributed to the development of FDA-approved oncolytic virotherapy against herpes simplex virus known as (TVEC or Imlygic), which limits virus replication and regulates human immunity, and which is used for treating human melanoma [63]. Following this approach, novel vaccination drives are being tailored in medical science to reduce the side-effects and induce long-term immunity against HSV infection, with the aim of achieving prophylactic and therapeutic goals to reduce viral infection and reduce the disease symptomology [43,62]. Moreover, efforts are being directed to reducing the neurotropism and latency associated with HSV while designing the live-attenuated vaccination regimens. Thus, by introducing certain insertions and/or or deletions in the viral progeny, the vaccination attempts show disrupting neuronal retrograde transport and the respective inability of HSV to affect neuronal cells [32,38,65]. Some important clinical ongoing trials in this regard are provided in Table 1.

#### 3.3.4. Peptide Vaccines against HSV

Peptide vaccines are developed on the principle that a single molecular entity or peptide epitope could generate massive immune responses to protect against a particular disease. In this regard, immunization with immuno-dominant T-cell epitopes or neutralizing epitopes has been tested and found to be protective [58]. This system of vaccination has shown better outcomes upon the combined application of certain adjuvants such as heat-shock proteins that may be expressed in recombinant viruses or bacterial expression systems [37]. However, the complications and limitations associated with the widespread human population and differential immune responses that may entail immunodominant responses by a certain peptide hinder the development of peptide-based vaccines [25,31]. However, efforts are still in research annals to develop better vaccination options for both serotypes of HSV.

#### 3.3.5. Killed-Virus Vaccines against HSV

Similar to the live-attenuated mechanism, this mechanism involves variations in terms of killed virus vaccination to avoid the risk of reactivation of viruses in subjects. Traditionally, phenol chemicals and UV light treatments have been used for this purpose, but other methods of viral inactivation have also been used more recently [79,80]. This approach is used as immunotherapy but remains underrated as it only provides little help to regress the viral infection, which is a property of natural infection. Recent advances have been made, and some newer studies are in progress that use sonication, chemicals, radiation, UV light, formaldehyde treatment, or their combination to cause viral death [80,81,82]. Moreover, experiments are performed by regulating the dosage amount, time, route, and number of administrations and combination with adjuvants to check the efficacy. However, further work is necessary to deduce the efficiency of this vaccination method [80,83].

#### 3.3.6. Fractionated-Virus Vaccines against HSV

In these protocols, HSV vaccines are prepared by subjecting the infected cultured cells to various procedures, which inactivate the virus particles while partially purifying some viral protein subsets [82,84]. Trials are ongoing on such vaccination methods. In simple terms, viral characteristic proteins such as those used in peptide vaccines (e.g., gPs) are mixed with inactive virus particles and with some adjuvants to produce a binding effect of the vaccine. Previous studies have shown little or no effect on immune responses; thus, this approach requires further work to induce productive clinical outcomes [30,31].

#### 3.3.7. Discontinuously Replicating Virus Vaccines against HSVs

In this method, some important genes required for viral replication or transmission are either deleted or replaced with other genes. The method is mainly used to study the functionalities of different proteins; however, the same approach is often used for designing vaccines [22,73]. These viruses may undergo replication but are unable to further infect the cell because they are transmission noncompetent. Because of this effect, they are termed as discontinuously replicating viruses. They exhibit the property of inability to restimulate periodically to have a recurrent, peripheral lytic replication cycle [47,64]. They have been checked in animal subjects for creating strong immune responses, with some candidates entering clinical trials, as indicated in Table 1. However, further work is required for effective clinical improvement in vaccination implications.

#### 3.3.8. Replication-Competent Live-Virus Vaccines against HSV

As the name indicates, these vaccines exhibit the replication property of viruses but undergo certain insertions and or deletion of encoded genes for application. They generate broad-scale immunostimulatory effects, including reactions from T and B cells and neutralizing antibodies. [85] They undergo the presentation of a complex mixture of epitopes with only a few missing genes. In the case of latency and reactivation from virus progeny, an endogenous re-boosting effect is created [12]. However, the limitation is that the possibility of mutation and reactivity with the wildtype strain of HSV in an immunocompromised individual may alter the vaccine mechanism. Moreover, complications may also be faced in terms of viral strain production and serological testing of HSV infection [12,13]. Several genetic studies have been conducted to understand which genes can be deleted for the preparation of replication-competent vaccines. This method is similar or identical to the method used in live-attenuated vaccination [61,63].

### 3.4. Therapeutics and Antiviral Strategies against HSV

When HSV infection was initially identified as a health concern, several therapeutic trials were put into research trials for evaluating different drugs against it. So much research was conducted around the time of the discovery of acyclovir back in the 1980s [21]. The search has not stopped even now, and new therapeutics are being developed that focus on different mechanisms of antiviral action [86,87,88]. This may include various approaches such as virus entry inhibitors, fusion, or virus-release inhibitors. Among these trials, N-docosanol (an entry inhibitor) is the only FDA-approved drug that is used to counter herpes labialis but not recurrent genital herpes or ocular infection [48]. More effective therapies are required to contain the global burden associated with HSV infections. Some of the major drugs with varying mechanisms of action are briefly described in the next section and a summary has been presented in form of Table 2 at the end of this section. 

#### 3.4.1. Receptor Targeting Therapeutics against HSV

These therapeutics work by preventing the receptor virus binding phenomena by targeting HSV entry molecules/receptors or glycoproteins on the host cell surface. They demonstrate both prophylactic and therapeutic efficiencies against HSV [38,47,89].

##### Anti-Heparan Sulfate Peptides

Two important receptor peptides, G1 and G2, have a role in binding to the cell surface receptors of HS (present in almost all cell types) and targeting them to block HSV-1 infection [55]. This phenomenon has been dose-dependently checked in cell line-based experiments. These results indicate the potential benefit of the inhibition of viral replication and cell-to-cell viral spread [56]. Similarly, experiments on animal models exhibited their prophylactic properties against ocular and genital infections. The overall number of genital lesions was reduced in tested subjects. However, a limitation of these drugs is the presence of HS receptors on all cells; thus, the drugs may produce side-effects in healthy cells, while there is a need to prevent the associated cytotoxicity [11,55,56].

##### Apolipoprotein E

Apolipoprotein E (apoE) is a glycoprotein that helps in viral attachment and entry by binding directly to heparin sulfate proteoglycans in the extracellular matrix of the host cell membrane [90]. Specifically, the tandem repeat dimer peptide, apoEdp, exhibits antiviral activity against both HSV 1 and HSV 2, as well as HIV. The effective results of these drugs have been shown to induce corneal infection along with immunomodulation in terms of downregulated proinflammatory and angiogenic cytokines [40,90]. Moreover, the drugs exhibited low or no systematic toxicity in mouse models. Their effect has been comparatively evaluated to be the same as that of the currently in-use drug trifluoro thymidine (TFT) against HSV-1. The therapeutic effects have largely been shown to reduce infection symptomology in animal models [40,90].

##### AC-8-Potential Cationic Peptide

AC-8 is an igG FAB fragment that exhibits antiviral properties by binding to the glycoprotein D receptor [57]. This drug has shown efficacy in terms of reducing corneal vascularization and keratitis in mouse models. This property is produced due to the essential role of Gd in the herpes virus entry mechanism, which AC-8 successfully targets to prevent a subsequent infection. It also reduces cytotoxicity and inflammation even after repeated usage [25,37,56].

#### 3.4.2. Nucleic Acid-Based Molecules

##### Aptamers

Aptamers are compounds that can bind with targeted molecules with a high affinity. They have characteristic features similar to antibodies; they fold in a different sequence-specific conformation determined by the target agents [91]. Several aptamer compounds have been proposed as antiviral agents in different infectious diseases, including HIV, cytomegalovirus, and recently against glycoproteins of HSV viruses [61]. RNA aptamers are major candidates under study that exhibit the antiviral potential to neutralize HSV species. Their highly specific nature allows scientists to define and manufacture specifically targeted aptamers that do not show a reaction against other viruses [61,91,92].

##### Dermaseptins

These form a family of associated poly cationic peptides derived from frog species. They exhibit antiviral properties against HSV species [93]. They interfere with the virus–host interaction owing to the positively charged amino acids that bind with the opposing negative charged heparin sulfate molecules of host cells [36,55,56,93]. Experiments showed they were effective against acyclovir-resistant HSV-1 species and had a reduced cytotoxic profile. They work well at low pH levels, which may allow them to remain active in the genital tract [55,56]. Some important cation ion peptides belonging to dermaseptins are indicated in Table 2.

#### 3.4.3. Viral Glycoprotein Targeting Therapeutics

As explained earlier, virus surface glycoproteins play an important function in fusion and viral entry into the host cell. They are positively charged molecules; hence, polyanionic compounds with negative charges could be designed and used to inhibit HSV fusion and replication in vitro by targeting the glycoprotein/sulfate compound complex [27,30,31,32]. Some important polyanionic compounds that have been used in research experiments are described briefly below.

##### Nanoparticles with Affinity to Bind GPs

Recent advances in nanotechnology-based therapeutics have presented newer methods for tackling viral infections. Hence, various experiments have been designed that may inculcate the properties of metallic nanostructure-based compounds with high affinity to bind viral glycoproteins [94,95]. As the virus binds to the HS with its surface gPs, a strategy could be devised simply by targeting the gPs. Some important nanoparticle species such as gold nanoparticles (AuNPs), tin oxide (SnO), zinc oxide (ZnO), mercaptoethane sulfonate (Au-MES), and some other important species are under research [94,95,96,97]. Moreover, the latest studies have demonstrated dual effectivity in terms of viral fusion inhibition and immune stimulation to protect against viral diseases. The overall effect is reduced virus entry, replication, transmission, mutation, and highly induced immune response against these virus infections. Moreover, the conjugation with other drugs and adjuvants may also provide added value to antiviral therapeutics [94,96].

##### K-5 and SP-510-50 Compounds

Since the presence of HSV-2 infection increases the likelihood of catching HIV-1 infection, therapeutics are being designed for a combined and simultaneous effect against both. In this regard, polyanionic K-5 compounds present a major therapeutic option to address this issue [30,31]. They work by inhibiting free virion infection by interfering with GPs and subsequently preventing cellular cross-transmission in vitro. With more advanced clinical experimentation, these compounds could be used against the sexual transmission of HSV and HIV diseases [48]. Similarly, SP-510-50 works as an antibody toward the gD of virus particles and, thus, provides antiviral infectivity in HSV patients. Their effect is bound to their dosage applicability for infection prevention [85,89]. They exhibited twofold better results compared to the commercial trifluoridine (TFT) using a lower dose. Moreover, the overall disease symptomology was reduced by their application [38].

##### Dendrimers

Dendrimers are composed of an amino-acid or carbohydrate conformation that is arranged in macromolecular compositions. Like nanoparticles, they exhibit good antiviral activities for their size [58]. Moreover, their characteristics, such as ease of preparation, ability to display a wide variety of surface molecules, easier functionalization, and targeted effect against viral gPs and the host cell surface make them an important therapeutic candidate for HSV treatment [31]. The surface characteristics make them eligible to bind multiple drug regimens, with a high and multidrug payload. Their successful application against HSV is in research annals. The purpose of these trials is to properly establish the safety, tolerability, toxicity, and systematic pharmacokinetic properties of these agents [31,58]. Some important ongoing trials are shown in Table 2.

#### 3.4.4. Targeting the Downstream Signaling Cascades

Targeting various downstream molecules that conduct cell signaling to induce viral infection is an important strategy that has been the focus of cell biology and bioinformatics recently. These studies allow the exploration of wide-spectrum molecular entities that could be used to design targeted therapies [98]. For example, studies have demonstrated the mechanism of different viruses that use actin and myosin-dependent pathways for the internalization of viruses in the cell [99]. The same property is exhibited by HSV which is involved in phagocytic uptake by corneal fibroblasts and retinal epithelial cells [98,100]. The underlying mechanisms are controlled by various kinases such as cyclic AMP-dependent protein kinase A, Akt/PKB, and ribosomal kinases p70 and p85, which play important roles in establishing cellular fusion [98,99,101]. Thus, inhibitor therapies are being designed against PI3K kinases to regulate the cellular surfing, entry, and viral infection in targeted cells. Successful results have been acquired in vitro, while next-level studies are still ongoing.

### 3.5. Antimicrobial Peptides against HSV

Antimicrobial peptides (AMPs) are positively charged short oligopeptides found in virtually all organisms which exhibit diversity in structure and function. They are synthesized and processed to play a vital role in initial immune responses against injury and infections. Some examples of such AMPs in humans include defensins, transferrins, hepcidin, cathelicidins, human antimicrobial proteins, histones, AMP-derived chemokines, and antimicrobial neuropeptides. AMPs have widely been studied for their potential antiviral properties. Defensins have been shown to play a protective role against HSV by blocking virus entry and other stages of the virus life cycle [102,103]. Several studies have shown a vital role of AMPs against various viral infections; therefore, AMPs can be effectively used as excellent therapeutic agents against HSV [104].

### 3.6. Some of the Latest Therapeutic Options

#### 3.6.1. Compounds Derived from Marine Resources (Algal Species)

The widespread HSV positivity in the human population has compelled the scientific community to continuously remain engaged in proposing different therapeutic regimens against HSV infections [21,46,48]. The traditionally used drugs such as acyclovir, ganciclovir, valaciclovir, and foscarnet are good options for HSV treatment; however, the development of drug resistance in patients and the ability for viruses to develop a mutation in strains has compelled scientists to look for other options [105]. Marine-based products, such as those derived from algal populations, bacterial species, fungal biomass, sponges, tunicates, echinoderms, and mollusk seaweeds, are important organisms from which these drug candidates are being derived [105]. Caulerpin is one of such candidate drugs that has its origin in marine algae and works well as an antioxidant, antifungal, antibacterial agent, and acetylcholinesterase (AChE) inhibitor [106]. It functions to inhibit the stages of the replication cycle [107]. Moreover, its application as an alternative to traditional acyclovir is under consideration. In addition to caulerpin, various other algal species (~40) are in research and development for exhibiting anti-HSV properties in resistant infections. They exhibit antiviral activity ranging from 50% to 80% for both species of HSV [21,46,48,105,106]. Different algae with antiviral properties are shown in Table 2. These studies allow the scientific community to delve deeper into marine-based and plant-based products to find a cure for HSV.

#### 3.6.2. Mucus Penetrating Particles

Since mucus formation is an unfortunate characteristic of the common summer cold, concurrent HSV and common cold infections could present a hurdle in drug delivery and penetration of the targeted cells [95]. Owing to the mucoadhesive characteristics exhibited by common drugs, some studies have been conducted to design mucous penetrating particles mainly based on nanoparticles. These neoformations easily penetrate the tissues of the sinuses and vagina and, thus, establish successful delivery of drugs to tissues of interest [95,96]. Moreover, they provide the opportunity to surface coat the particles with multiple antiviral drugs and enable better absorption of the nanosized particles for a more profound effect. Overall, MPPs improved drug binding, distribution, retention, and dosages, as well as reduced toxicity, in HSV model experiments [95,96,108].

#### 3.6.3. Plant-Derived Therapeutic Options

Similar to algal-derived drug candidates, some recent studies have indicated the therapeutic potential of some plant-based products (Table 2). Like other drug regimens, they inhibit the virus entry and replication cycle by acting as potent inhibitors of various glycoproteins specific to different antiviral plant agents [109]. Antiviral agents such as neem bark extract (NBE) derived from Azardirachta indica and cyanovirin-n (CV-N) derived from Nostoc ellipsosporum, as well as peri-acylated gossylic nitriles derived from gossypol, are some of the important drug candidates exhibiting efficient anti-HSV profiles [42,47,48,109,110]. However, the potent anti-HSV profiling, toxicity studies, pharmacokinetic profiling, and antidrug comparative studies remain to be conducted in detail to provide the benefits associated with plant-based herbal therapies [109].

#### 3.6.4. Combined Therapies

Knowing the scope of HSV disease implications, scientists are now gathering their research focus toward combined therapies since a certain specific drug or vaccine has not yet been shown to eradicate HSV infection [21,30,48,61]. Therefore, more integrated and coordinated efforts are being put forth in the form of combined therapies, where several drug combinations are checked for their effect against HSV. Most of the individual drug regimens have already gone through scientific examination to establish their antiviral character. Hence, the purpose of combined therapies is to only evaluate multiplex combined antiviral effects against HSV infection [46,48]. Various experiments in research annals have been carried out in vitro, in animal models, and in clinical trials. Similarly, more specific studies are in the research phase against proven anti-HSV drugs such as acyclovir and acycloguanosine in terms of evaluating their cytotoxic and pharmacokinetic profiles and upgrading them by nano-scaling or conjugating with nanoparticle formulations for effective low dosage implications [59,94,95,96,97]. These latest studies have provided a doorway to the resistance that develops over time in patients. The new formulation offers lower dosage, more targeted delivery, and enhanced efficacy in tested subjects. Therefore, the field of combined therapy against HSV is a major player in the future drug and vaccination designs against HSV. A brief overview of these therapeutic strategies against HSV have been covered in a summarized version in Table 2 below.

**Table 2:** Ongoing trials for HSV drugs.

Sr. No.	Drug Type	Ongoing Trials	Refs.
1.	Receptor-targeting therapeutics	G1 and G2 anti-heparan sulfate peptides Apolipoprotein E AC-8 Aptamers (against enveloped gD glp (HSV-1 and HSV-2), Dermaseptins (group of lysine-rich peptides S1–S5 and K4K20S4, indolicidin, melittin, cecropin A, magainin I and II, and indolicidin)	[27,31,46,47,58,89,93,111]
2.	Viral glycoprotein-targeting therapeutics	Nanoparticles (ZnO and SnO), protein microspheres (PM), AuNPs capped with (Au-MES) K-5 Compounds-(*E. coli* derived K5 polysaccharides including K5-N,OS(H), and Epi-K5-OS(H)) Polyionic compounds (SP-510-50, PRO-2000, cellulose sulfate, poly-methylene hydroquinone sulfonate, and polystyrene sulfonate) Dendrimers (glycodendrimer and peptide-dendrimers), such as SPL7013 Dendrimer with peptide gH625 Polycationic dendrimers: SB105 and SB105_A10	[31,38,46,47,58,89]
3.	Targeting cellular signaling cascades	PI3K family of heterodimeric enzymes inhibitors Akt/PKB inhibitors Cyclic AMP-dependent PKA inhibitors Inhibitors of PKC isoforms Inhibitors of ribosomal S6 kinases p70 and p85	[98,99]
4.	Marine organism-derived therapeutics	Caulerpin from *Caulerpa Lamouroux* (Caulerpales) Rhodophyta (16 species) Ochrophyta (8 species) Chlorophyta (12 species) Green algal species: *Ulva fasciata* and *Codium decorticatum* Red algal species: *Laurencia dendroidea*	[105,106]
5.	Mucus-penetrating nanoparticles	Coated polystyrene/biodegradable poly (lactic-*co*-glycolic acid) with pegylated (PEG) NPs MMPs + acyclovir (ACVp-MPPs) Plant-derived antiretroviral *Momordica charantia* (proteins MAP30) *Gelonium multiflorum* (proteins GAP31) Gossypol (from cottonseed oil) and peri-acylated gossylic nitrile derivatives	[95,96,108]
6.	Combinations trials on drugs	Trifluridine + idoxuridine + vidarabine Trifluridine + vidarabine Trifluridine + acyclovir Brivudine + idoxuridine Brivudine + trifluridine Brivudine + acyclovir Ganciclovir + acyclovir Foscarnet + trifluridine Foscarnet + acyclovir Foscarnet + ganciclovir Antiviral + interferon Debridement + antiviral	[22,30,45,46,48,79,106,111]
7.	Other anti-therapies compounds in research trials	*E*-5-(2-Bromovinyl)-2′-deoxyuridine *E*-5-(2-Iodovinyl)-2′-deoxyuridine 5-Vinyl-2′deoxyuridine 2′-Fluoro-5-iodoaracytosine Acycloguanosine and 5-iodo-2′-deoxycytidine, Acycloguanosine (WELLCOME 248U)-(9-[2hydroxyethoxymethyl]guanine)	[20,109,110,111]

## 4. Conclusions

Several vaccines and drug trials are in progress against HSV. They provide a promising therapeutic potential in individual studies. However, no profound and specific therapy has been established until now that could tackle the problem of HSV infection worldwide. The need is to establish more coordinated and integrated studies with the cooperation of scientists, doctors, and pharmacies to take drug testing one step ahead in clinical practice. This is important because the expected viral mutations present the threat of the development of another mutant HSV that could then become another complication for HSV treatment and prevention. Therefore, the most effective approach for future therapeutic development will be to develop modern drug-design approaches such as those based on plant products and nanotechnology, and to carry out more combined therapies for large-scale and broad-spectrum antiviral and immunostimulatory effects so that HSV complications can be successfully addressed in the coming years.

## Figures and Tables

**Table 1:** An update on current vaccine candidates against HSV.

Sr. No.	Vaccination Protocol	Vaccine Candidates under Trial	Refs.
1.	Subunit vaccines	GEN-003 (gD2/ICP4 + matrix M2 adjuvant) gD2/gC2/gE2 (glycoprotein target) Monovalent gD2 vaccine and gD2 + alum Subunit HSV-2 Bivalent vaccine containing (gD2 and gB2) + nanoemulsion NE01 adjuvant Bivalent vaccine + MF59 adjuvants Subunit HSV-2 trivalent vaccine containing (gC2, gD2, and gE2) + CpG (5′-TCCATGACGTTCCTGACGTT-3′)/alum Chiron vaccine containing gB2 and gD2 (with deletions at amino acid 696 and 302 respectively) + adjuvant MF59 and MTP-PE Adjuvant MF59 alone	[42,50,51,52,53]
2.	Peptide vaccines	HerpV + (HSP + 32–35-mer peptides + QS-21 adjuvant and heat-shock proteins) Vaccine based on immune-dominant CD8 and CTL neutralizing epitope T-helper epitope adjuvants Heat-shock protein adjuvants HLA (A*0201)-restricted epitope in monovalent gB2 + HSP adjuvant	[25,27,31,37,54,55,56,57,58]
3.	DNA vaccines	Codon optimized polynucleotide vaccine: gD2 codon + tagged ubiquitin gD/UL46, + Vaxfectin^®^ adjuvant Polyvalent HSV-2 vaccine containing glycoproteins (g) B2, C2, D2, E2, H2, L2, and I2 + IL-12 adjuvant Nucleoside-modified mRNA encoding gC2, gD2, and gE2 + lipid nanoparticles (LNP) (MVA) vector encoding glycoprotein (gD2) VCL-HB01/HM01	[28,29,59,60,61,62]
4.	Live attenuated or replication deficient virus-based vaccines	HSV529 (HSV-2 having deletions in UL5 and UL29) ΔgD2 (HSV-2 with deleted Gd2) HF10 (HSV-1 with mutations in regions UL43, 49.5, 55, UL56, and LAT) HSV-2 0ΔNLS (HSV-2 with deletion of ICPO) RVx201 (derivative of HSV-2 0∆NLS) AD472 (HSV-2 with mutations in g34.5, UL43.5, 55–56, US10, 11, and US12) Gd2 dominant neg HSV-2 (CJ2-gD2) (HSV-GS3 and HSV-GS7) SP0148 (ACAM/HSV 529), replication-deficient HSV-2 (with deletions in regions UL5 and UL29) VC2-HSV-1 vaccine (with deletions in region of gK aa31-68 and UL20 aa4-22) RVx1001 (HSV-1 VC2) R2 non-neuroinvasive HSV-1 vaccine (HSV1-GS6264, 5 missense mutations in UL37) gH-deleted HSV-2 vaccine HSV-2 DISC Thymidine kinase-deficient mutants of HSV-1 and HSV-2 RAV 9395 with deletions of UL55 and UL56 Strain R7020, with deletions extending from UL54 (encoding ICP27) via promotor ICP4 replaced by gD2, G2, I2, and a part of gE2.	[32,38,43,62,63,64,65]
5.	Prime-pull strategy	live attenuated HSV-2 + topical intravaginal CXCL9/CXCL10 chemokine activation Epitopes based on CD8 + T-cell peptide (UL44 aa400–408, UL9 aa196–204, and UL25 aa572–580) + adjuvant CpG (Prime) and AAV8 vectored CXCL10 (Pull)	[32,38,64,65]
6.	Inactivated vaccine candidates	Formalin inactivated HSV-2 + MPL/alum	[66]
7.	Viral vector agents	gB1 of HSV-1 expressing Lentivirus vector gB1s-NISV (recombinant HSV-1 Gb_+_ intranasal nonionic surfactant vesicles) Plasmid based vaccine VCL-HB01 encoding HSV-2 proteins + Vaxfectin	[25,67,68,69,70,71]

## Data Availability

Not applicable.

## Abbreviations

AChE	Acetylcholinesterase inhibitor
Akt/PKB	Protein kinase B
apoE	Apolipoprotein E
CDC	Centers for Disease Control and Prevention
FDA	Food and Drug Administration
GP/gPs/gps	Glycoproteins
HIC	High-income countries
HS	Heparan sulfate
HSV	Herpes simplex virus
HSV-1 and HSV-2	Herpes simplex virus type 1 and type 2
igG	Immunogloblins
Au-MES	Mercaptoethane sulfonate
AuNPs	Gold nanoparticles
LMIC	Low-income countries
MMPS	Mucus-penetrating particles
NPs	Nanoparticles
ORFs	Open reading frames
PI3K	Phosphoinositide 3-kinases
PKA	Protein kinase A
PKC	Protein kinase C
PM	Protein microspheres
R&D	Research and development
SnO	Tin oxide
WHO	World Health Organization
ZnO	Zinc oxide
